# De Novo Development of mtDNA Deletion Due to Decreased POLG and SSBP1 Expression in Humans

**DOI:** 10.3390/genes12020284

**Published:** 2021-02-17

**Authors:** Yeonmi Lee, Taeho Kim, Miju Lee, Seongjun So, Mustafa Zafer Karagozlu, Go Hun Seo, In Hee Choi, Peter C. W. Lee, Chong-Jai Kim, Eunju Kang, Beom Hee Lee

**Affiliations:** 1 Department of Convergence Medicine and Stem Cell Center, Asan Medical Center, University of Ulsan College of Medicine, Seoul 05505, Korea; yeonmilee82@gmail.com (Y.L.); leemiju082@gmail.com (M.L.); soseongjun7@gmail.com (S.S.); zaferka@gmail.com (M.Z.K.); 2 Medical Genetics Center, Asan Medical Center, University of Ulsan College of Medicine, Seoul 05505, Korea; kth860828@gmail.com (T.K.); tjrhgns610@gmail.com (G.H.S.); ina@amc.seoul.kr (I.H.C.); 3 Department of Biomedical Sciences, Asan Medical Center, University of Ulsan College of Medicine, Seoul 05505, Korea; pclee@amc.seoul.kr; 4 Department of Pathology, Asan Medical Center, University of Ulsan College of Medicine, Seoul 05505, Korea; ckim@amc.seoul.kr

**Keywords:** mtDNA deletion, POLG, SSBP1, Pearson syndrome, human

## Abstract

Defects in the mitochondrial genome (mitochondrial DNA (mtDNA)) are associated with both congenital and acquired disorders in humans. Nuclear-encoded DNA polymerase subunit gamma (*POLG*) plays an important role in mtDNA replication, and proofreading and mutations in *POLG* have been linked with increased mtDNA deletions. *SSBP1* is also a crucial gene for mtDNA replication. Here, we describe a patient diagnosed with Pearson syndrome with large mtDNA deletions that were not detected in the somatic cells of the mother. Exome sequencing was used to evaluate the nuclear factors associated with the patient and his family, which revealed a paternal *POLG* mutation (c.868C > T) and a maternal *SSBP1* mutation (c.320G > A). The patient showed lower *POLG* and *SSBP1* expression than his healthy brothers and the general population of a similar age. Notably, c.868C in the wild-type allele was highly methylated in the patient compared to the same site in both his healthy brothers. These results suggest that the co- deficient expression of *POLG* and *SSBP1* genes could contribute to the development of mtDNA deletion.

## 1. Introduction

Mitochondria are cellular organelles that are surrounded by a double membrane, which is found in almost all eukaryotic cells. They contain a circular genome, mitochondrial DNA (mtDNA), which encodes proteins, rRNAs, and tRNAs necessary for mitochondrial function and exhibits maternal inheritance patterns [1]. Mitochondria play an essential role in energy production by producing adenosine triphosphate (ATP). They have been implicated in many physiological processes, including the production of reactive oxygen species (ROS), pyrimidine and lipid biosynthesis, regulation of cellular substrate levels, apoptosis, metal metabolism, calcium homeostasis and flux, neurotransmitter synthesis, heat production, and insulin secretion [2].

Defects in mtDNA have been linked to multiple human disorders, including multisystem congenital diseases and acquired degenerative disorders [3]. Additionally, they have been related to human aging [4] and are deemed the genetic cause of various human diseases, such as Pearson syndrome (PS), progressive external ophthalmoplegia (PEO), Kearns–Sayre syndrome (KSS), and Leigh syndrome [5,6]. The common mtDNA defect is a specific 4977 bp (mt8468–13446) deletion, which has been identified in various diseases and tissues associated with aging [7,8]. Similar to this deletion, most mtDNA deletions are seen in the major arc between replication origins of heavy and light strands (O_H_ and O_L_, respectively) [9] and predominantly flanked by short direct mtDNA repeats. Mainly, these deletions tend to develop during the repair of damaged mtDNA rather than replication [9,10].

The genes required to regulate and facilitate mitochondrial function are encoded by both mitochondrial and nuclear DNA, which include 37 mtDNA genes and more than 1000 nuclear genes [11]. Among the nuclear genes, the DNA polymerase subunit gamma (*POLG*) encodes a catalytic subunit of the mitochondrial DNA polymerase, which plays an important role in mtDNA replication and proofreading [12,13]. Mutations in *POLG* are associated with the development of mtDNA point mutations and various large mtDNA deletions in different somatic tissues [6,13,14]. Additionally, a previous study using transgenic *Polg* mutator mice could evidence the link between POLG expression and mtDNA deletion [15].

SSBP1 is also an important protein for mtDNA replication. It must stabilize single-stranded mtDNA and stimulate DNA synthesis by POLG [16]. *SSBP1* mutation impairs the amount of mtDNA and its replication [17]. 

In this study, we described a patient diagnosed with PS who exhibited a large deletion in his mtDNA. Noteworthy, this large deletion was not detected in maternal somatic cells. Furthermore, the association between *POLG* and *SSBP1* expression and de novo mtDNA deletion was investigated, thus gaining further insight into the contributions of the nuclear genome in the development of mtDNA defects.

## 2. Materials and Methods

### 2.1. PCR Assay for Large mtDNA Deletions 

DNAs were extracted from multiple tissues of both the PS patient and selected family members. The region surrounding the mtDNA deletion site was amplified by PCR using the following primer sets designed to detect major deletion and wild mtDNA in PS: mt8249F GGGCCCGTATTTACCCTATAG, mt9028R GGCCTGCAGTAATGTTAGCG, and mt13737R GAGAAATCCTGCGAATAGGC. PCR reactions were performed under the following conditions: 1 cycle at 94 °C for 5 min; then 35 cycles at 94 °C for 30 s, 56 °C for 20 s, and 68 °C for 1 min; followed by 1 cycle at 68 °C for 3 min. To detect multiple mtDNA deletions, the following primer sets were used: mt3163F GCCTTCCCCCGTAAATGATA and mt14342R GGGTGGTGGTTGTGGTAAAC. PCR reactions were performed under the following conditions: 1 cycle at 94 °C for 5 min; then 35 cycles at 94 °C for 30 s, 56 °C for 20 s, and 68 °C for 30 s, 1 min 30 s, and 2 min 30 s; followed by 1 cycle at 68 °C for 3 min. 

### 2.2. Quantitative PCR for the Detection of mtDNA Deletions

Heteroplasmy of the mtDNA deletions was evaluated using a qPCR assay. DNA was extracted from multiple tissues, and regions of the mtDNA, including deletions (mt8921–13316) in the PS patient, were amplified by PCR using the following primer set: F-8822 CTATAAACCTAGCCATGGCC and R-13436 GGTATGGTTTTGAGTAGTCC. PCR reactions were performed under the following conditions: 1 cycle at 94 °C for 10 min, then 40 cycles at 95 °C for 15 s, and finally 1 cycle at 60 °C for 1 min. Common mtDNA regions were also amplified using PCR with the following primer set: F-16483 GTGAACTGTATCCGACATCTG and R-100 CAGCGTCTCGCAATGCTATC. The PCR reaction conditions used here were identical to those used for the mtDNA deletion PCR. Heteroplasmy for each deletion was calculated as follows: heteroplasmy of deletion (%) = (quantity of deletion mtDNA/quantity of common mtDNA) × 100.

### 2.3. Skin Fibroblast Isolation and Culture, and Single-Cell Sub-cloning 

A skin biopsy sample was taken from the upper arm and dissected into small pieces, incubated in 0.1% collagenase (type IV) for 30 min, and plated into 60 mm dishes containing Dulbecco’s Modified Eagle Medium/Nutrient Mixture F-12 (DMEM/F12 medium, Life Technologies, Eugene, OR, USA) supplemented with 10% fetal bovine serum (FBS; Life Technologies) and 4 ng/mL of basic fibroblast growth factor (bFGF; Sigma, Saint Louis, MO, USA). For single-cell sub-cloning, 500 cells were plated in 100 mm dishes, confirmed, and marked for every single cell at a sufficient distance from another cell. These single cells then grew into colonies that were randomly selected and re-plated in six-well culture dishes, cultured, and then subjected to DNA extraction. 

### 2.4. Mitochondrial Respiration 

Mitochondrial respiration was measured using the XF Cell Mito Stress Test kit in an XF24 Extracellular Flux Analyzer (Seahorse Bioscience, Billerica, MA, USA) according to previously described methods [18]. The oxygen consumption rates (OCRs) for the mitochondria were measured using a serial addition of oligomycin (0.5 µg/mL) for ATP production (Subtraction basal OCR from oligomycin OCR), carbonyl cyanide 4-(trifluoromethoxy) phenylhydrazone (FCCP, 1 µM) for maximal respiration and reserve capacity (Subtraction basal OCR from maximal OCR), and antimycin A (1 µM) and rotenone (0.5 µM) for non-mitochondrial oxygen usage. These values were then normalized against baseline oxygen consumption. 

### 2.5. Exome Sequencing

Nuclear whole-exome sequencing (WES) was performed using peripheral leukocytes of the subjects in this study by using the SureSelect V6+UTR-post panel (Agilent Technologies, Santa Clara, California, USA) and the HiSeq 2500 platform (Illumina, San Diego, California, USA) according to previously described methods [19] with minor modifications. The exomes were captured using the SureSelect V6+UTR-post panel (Agilent Technologies) and sequenced on a HiSeq 2500 platform (Illumina). Raw sequencing data from WES were aligned to the reference genome hg19 using the Burrows–Wheeler Aligner (BWA v0.7.17; http://bio-bwa.sourceforge.net/, accessed on 13 February 2021). Duplicates were marked with Picard tools (v2.18.29; http://picard.Sourceforget.net, accessed on 13 February 2021), and variants were called using the software package Genome Analysis Toolkit (v4.1.1.0; https://software.broadinstitute.org/gatk/, accessed on 13 February 2021). Functional annotations of all variants were assigned using ANNOVAR software (v4.3t; http://SnpEff.sourceforge.net/, accessed on 13 February 2021). All variants in the 3’ untranslated region (UTR), 5’UTR, upstream, and downstream introns and intergenic non-coding regions were filtered out. Then, synonymous and common variants with minor allele frequencies of ≥0.01% (1000 Genomes Project, the NHLBI GO Exome Sequencing Project (ESP6500 data set), and dbSNP142) were also filtered out. The pathogenicity of variants was assessed using in silico prediction tools, including Polymorphism Phenotyping v2 (PolyPhen-2; http://genetics.bwh.harvard.edu/pph2, accessed on 13 February 2021) and sorting intolerant from tolerant (SIFT; http://sift.jcvi.org, accessed on 13 February 2021).

### 2.6. Sanger Sequencing

The c.868 position in *POLG* and the c.320 position in *SSBP1* were amplified using PCR with the following primer set: *POLG* F-CCTGGAGGTCCCTACTGGTG and R-TGTCCTTCATGGTGCCCTTC and *SSBP1* F-GGGCTAGATCCTTCCAAAAG and R-CCGTAAGAGAAATAGCTGAC. PCR reactions were performed under the following conditions: 1 cycle at 94 °C for 5 min; then 35 cycles at 94 °C for 30 s, 56 °C for 20 s, and 68 °C for 30 s; followed by 1 cycle at 68 °C for 3 min. PCR products were purified, sequenced, and analyzed using Sequencher v5.0. 

### 2.7. Quantitative RT-PCR (qRT-PCR) for POLG and DNMT Expression

Total RNA was extracted using the RNeasy Mini kit (Qiagen, Hilden, Germany), and complementary DNA (cDNA) was synthesized using the SuperScript^TM^ VILO^TM^ cDNA Synthesis kit (Invitrogen, Carlsbad, California, USA) according to the manufacturer’s specifications. qPCR analysis was performed according to previously described methods [20] with minor modification using the following gene-specific primers: *POLG* F-CTCTTGACCAGGTGCATGTTTG and R-TGCACTGAAAAAGGCGACTG, *SSBP1* F-TCAGGACCCTGTCTTGAGA and R-GATATTCTGTGCCATGTTGTC, *DNMT1* F-CGAGGACGAAGATGGAGACG and R-CTGAATGCACTTGGGAGGGT, *DNMT3A* F-ATCTACGAGGTCCTGCAGGT and R-CACCCACATGTCCGTGTACA, *DNMT3B* F-TGCTCTTCCTCAGCTGTGTG and R-CTGTCGGCACTGTGGTTTTG, and *β-ACTIN* F-TGCTATCCCTGTACGCCTCT and R-CTCCTTAATGTCACGCACGA. The expression levels for each gene were measured by qRT-PCR using SYBR Premix Ex Taq (Takara, Kyoto, Japan). The reaction parameters for qRT-PCR were as follows: 1 cycle at 95 °C for 15 min; followed by 35 cycles at 95 °C for 10 s, 60 °C for 15 s, and 72 °C for 20 s; and a final elongation step at 72 °C for 5 min. Gene expression levels were expressed as relative 2^-ΔΔCt^ values.

### 2.8. Protein Preparation and Western Blot

Western blot was performed according to the previously described methods [21] with minor modifications. Samples were extracted following homogenization in RIPA buffer, and protein concentrations were determined using a protein assay kit (Bio-Rad, Hercules, California, USA) with BSA as the standard. A total of 10 μg of protein was subjected to electrophoresis using 10%–12% SDS-polyacrylamide gel. Then, these proteins were transferred onto a polyvinylidene difluoride (PVDF) membrane (Bio-Rad). The antigens were detected using primary antibodies (anti-POLG, diluted 1:1000, ab128899, Abcam; anti-SSBP1, diluted 1 μg/mL, AF6588, Bio-techne; anti-β-ACTIN, diluted 1:1000, ADI-CSA-335, Enzo; and anti-GAPDH, diluted 1:2500, ADI-CSA-335-E, Enzo), followed by secondary immunoglobulins conjugated to HRP (1:1000, Bio-Teche for SSBP1 and 1:10,000, GeneTex for POLG, β-ACTIN, and GAPDH), and an ECL reagent kit was used for visualization (pico EPD, Elpisbio, Daejeon, South Korea). The assay was triplicated, and the expression of the POLG protein was normalized with the β-ACTIN and GAPDH expression. The band intensities were completed using ImageJ software (National Institutes of Health, Bethesda, MA, USA).

### 2.9. Methylation Analysis by Pyrosequencing

DNA was isolated using the DNeasy Blood and Tissue kit (Qiagen) and bisulfite-treated using the EpiTect Fast DNA Bisulfite kit (Qiagen) according to the manufacturer’s instructions. Bisulfite-treated gDNA was amplified via PCR using the following primers: 

CpG island (c.502–613)−1: F-GTTAATTTGTTGTTGTAGGTTTAGTTG, R-biotin- AACTAAACCCCCATACCTACTTATA, and sequencing primer F-TGTTGTAGGTTTAGTTGT; CpG island (c.502–613)−2: F-biotin-GTTAATTTGTTGTTGTAGGTTTAGTTG, R-ACCACCCCCAATATAAAACAAATTCCC, and sequencing primer R-TCCCTCTACCAAACA; CpG sites (c.862 and 868): F-biotin-GAGGGGAGGTTGTTTGAGGA, R-ACCCTAAAATAACCATATACATACTCAT, and sequencing primer R-ATATACATACTCATAATATCCAAAA; and CpG sites (c.925 and c.1022): F-biotin-AGTATGTATATGGTTATTTTAGGGTTAAGT, R-biotin-AAATCCCAAACACTATACTCCTACCC, sequencing primer 1-F-TTATTTTAGGGTTAAGTAGTTTTTA, and sequencing primer 2-F-AGAGGAAAGTTAGAAGAG. PCR products were subjected to pyrosequence using the PyroMark Q48 Autoprep system (Qiagen) and were analyzed using PyroMark Q48 Autoprep 2.4.2 software (Qiagen) [22].

### 2.10. Statistical Analysis

The data are presented as the mean ± standard error of the mean (SEM). Independent-group *t*-tests for unpairwise comparisons or ANOVA with Tukey’s analysis for multiple comparisons were used to evaluate statistical significance (GraphPad Prism, San Diego, CA, USA), and a *p*-value of <0.05 was considered as significant.

## 3. Results

### 3.1. Pearson Syndrome (PS) with a Large mtDNA Deletion

**Figure 1:**
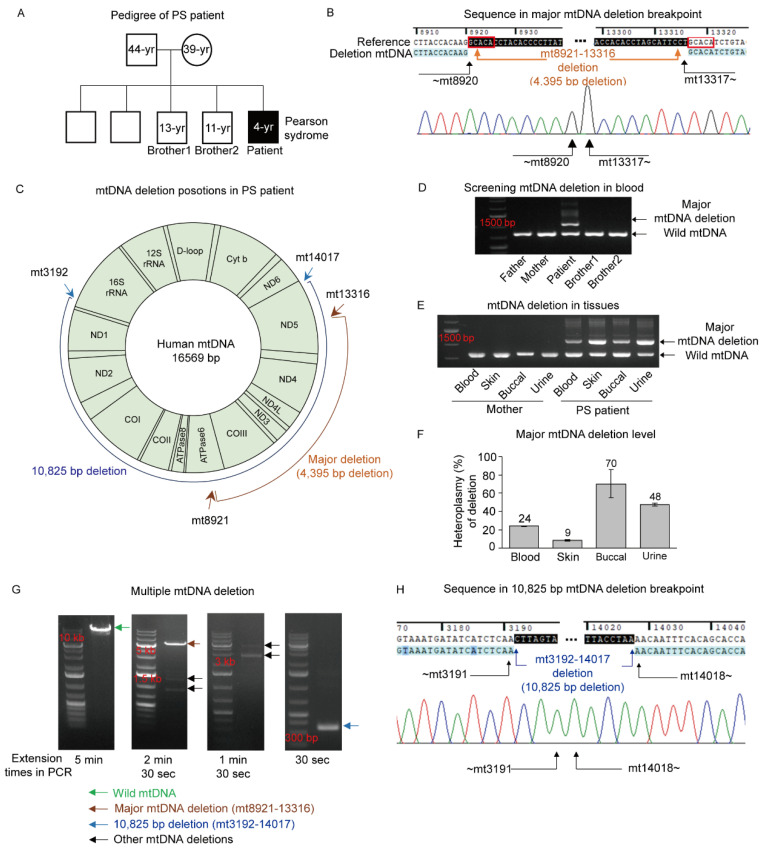
Mitochondrial DNA (mtDNA) deletions in a Pearson syndrome (PS) patient. (**A**) Pedigree of the PS patient: A male patient, diagnosed with Pearson syndrome, with healthy parents and four healthy brothers. (**B**) The breakpoint sequence around the 4395 bp deletion demonstrates that the deletion is flanked by 5 bp (GCACA) direct repeats (mt8921–8925 and mt13317–13321, red boxes). The automatic sequencing peak showing the breakpoints of the 4395 bp deletion spanning nucleotides 8921 and 13316 of the mtDNA. (**C**) Schematic of mtDNA deletions in the PS patient; major deletion, mt8921–13316; 10,825 bp deletion, mt3192–14017. (**D**) Screening for mtDNA deletion in blood samples from the PS patient and his family using PCR. PCR gel images showed that mtDNA deletion was detected only in the PS patient. Wild mtDNA represents the amplification of non-deleted mtDNA. (**E**) Detection of mtDNA deletion in various tissues from the PS patient and his mother using PCR. PCR gel images showed that the mtDNA deletion was detected only in the PS patient. Wild mtDNA represents the amplification of non-deleted mtDNA. (**F**) Heteroplasmy levels for the major mtDNA deletion (mt8921–mt13316) in multiple tissues from the PS patient. Each assay was completed in triplicate, and data are represented as the mean ± SEM. (**G**) Detection of mtDNA deletions. Multiple mtDNA deletions between mt3163 and mt14342 were detected at different PCR extension times. (**H**) The breakpoint sequence around the 10,825 bp deletion. Automatic sequencing peak showing the breakpoints of the 10,825 bp deletion spanning nucleotides 3192 and 14017 of the mtDNA.

The PS patient was the fifth child of a family with four healthy male children from non-consanguineous parents (Figure 1A). At 5 months of age, he presented with sideroblastic anemia, pancytopenia, and bone marrow failure. At 2 years of age, he was identified with proximal renal tubular acidosis (Table 1). When he reached the age of 3, he was affected by chronic kidney disease, exocrine pancreatic insufficiency, adrenal cortical insufficiency, and developmental delay. He was finally diagnosed with PS with mtDNA deletions in peripheral leukocytes. 

The mtDNA sequence analysis of the PS patient showed that the major deletion was 4395 base pairs (bp) located between *ATPase6* (mt8921) and *ND5* (mt13316) genes (Figure 1B,C). However, this deletion has not been previously recorded in the human mitochondrial genome database MitoMAP (https://www.mitomap.org, accessed on 13 February 2021). Nevertheless, in a manner similar to previous cases of deletion phenomena, short direct mtDNA repeats were detected in this novel deletion [10]. The repeats were 5 bp in length (GCACA) and placed at both ends of the deletion (mt8921–8925 and mt13317–13321, Figure 1B). Further investigation on mtDNA deletions in the blood cells of the PS patient and his family members revealed that the major deletion was exhibited only in the PS patient but not in family members (Figure 1D). For confirmation, multiple tissues from the PS patient were examined, including blood, skin, and both buccal and urine epithelia. As a result, a major deletion was detected in these tissues (Figure 1E). For investigating maternal transmission of mtDNA deletion, multiple tissues from the mother were also examined. However, no mtDNA deletions were detected in the maternal tissues (Figure 1E). Even so, there was a possibility that the mother had an extremely low heteroplasmic state or that the deletion had occurred in only a limited number of individual cells [18]. Accordingly, skin fibroblasts from the mother were isolated and evaluated. A total of 10 fibroblast colonies (FC) were selected at random and subjected to single-cell analysis. However, no individual colonies carried the deletion. Although the mother’s somatic cell was evaluated due to a lack of sample from the mother’s germ cells, these findings could not exclude the possibility that the patient’s mtDNA deletion was maternally inherited. The incidence of the major deletion in the PS patient and his family members was quantified within a 0.05% detection limit. Among the family members, only the PS patient harbored the mtDNA deletion and exhibited heteroplasmy between 9% and 70% in various tissues (Figure 1F). Additional examinations were performed to investigate other mtDNA deletions in the PS patient. We expected that the short direct repeats could be associated with multiple mtDNA deletions. Therefore, the area between mt3163 and mt14342 was explored because there were 15 sites of 5 bp direct repeats out of 17 sites. Multiple mtDNA deletion bands, including major deletions, appeared at different extension times during the PCR cycle (Figure 1G). Among the multiple bands, the band appearing in 30 s of extension time was selected because of the more abundant amplification than other bands, resulting in mtDNA deletion located between mt3192 and mt14017 (Figure 1H). This large deletion of 10,825 bp was place-encoded 16S rRNA through ND5 genes; however, this deletion was not flanked by direct repeats, unlike the major deletion (Figure 1H). 

**Figure 2:**
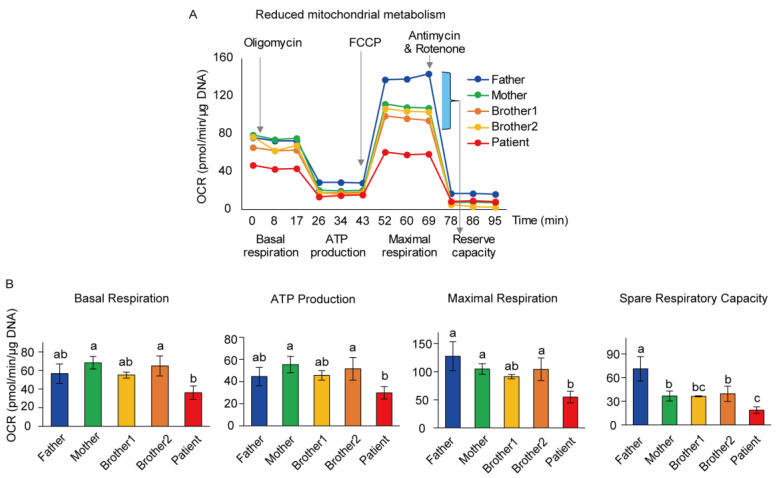
Lower mitochondrial metabolism in the PS patient. (**A**) Fibroblasts of the PS patient displayed lower mitochondrial oxidative capacity compared to those of family members. (**B**) The significantly lower level of mitochondrial metabolism in the PS patient. Basal respiration and ATP production were significantly reduced in the PS patient compared to the mother and brother 2. The maximal respiration and the spare respiratory capacity in the PS patient were significantly lower than in the father, mother, and brother 2. Each assay was performed in triplicate, and data are represented as the mean ± SEM. a, b, and c indicate significant (*p* < 0.05) differences among family members.

The oxygen consumption rate (OCR) is an indicator of mitochondrial respiration and energy production [18,23]. Hence, the OCRs of skin fibroblasts isolated from the PS patient and his family were measured and compared. Mainly, the OCR of the PS patient’s skin fibroblasts was lower than that of his family members (Figure 2A). The PS patient showed significantly lower basal respiration and ATP production compared to the mother and brother 2 (Figure 2B). Further, the maximal respiration and the spare respiratory capacity in the PS patient were significantly lower than those in other family members, except brother 1.

### 3.2. Identification of Nuclear POLG and SSBP1 Mutations by Exome Sequencing 

**Figure 3:**
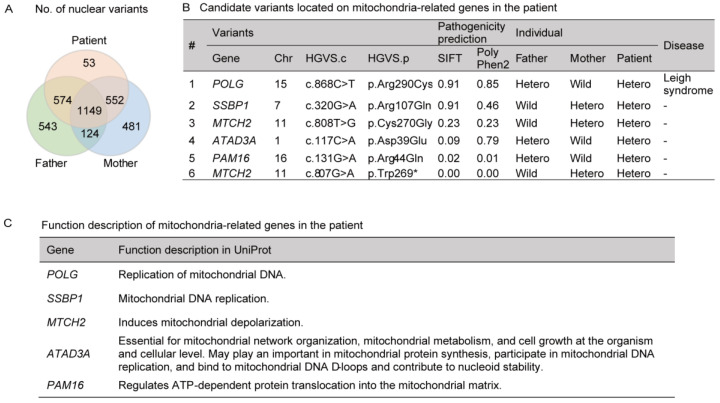
Candidate disease-causing variants from the PS patient identified by exome sequencing. (**A**) Venn diagram of the nuclear variants detected after exome sequencing of samples collected from the PS patient and his parents. A total of 2328 variants were detected in the PS patient. (**B**) Candidate variants were located in mitochondria-related genes in the PS patient. A total of six variants were selected as candidates for diseases with minor allele frequencies of <0.03 in the general population. Variants were listed in order of their pathogenicity prediction ranking. Polymorphism Phenotyping v2 (PolyPhen-2) and sorting intolerant from tolerant (SIFT) are in silico prediction tools for pathogenicity assessment. HGVS.c, Human Genome Variation Society, coding DNA reference sequence; HGVSs.p, Human Genome Variation Society, protein reference sequence; Hetero, heterozygous mutation; Disease, disease prediction based on the GATK analysis pipeline. (**C**) Functional description of the mitochondria-related genes in UniProt.

Since there was no deletion detected in the mtDNA of maternal somatic tissues, the nuclear genome’s relation with the major deletion was investigated. Nuclear exome sequencing was performed using the peripheral leukocytes of the PS patient and his parents. In comparison, a total of 2328 variants were detected in the PS patient, 53 of them de novo (Figure 3A). However, none of the unique variants was recorded to be related to the mitochondria in the Universal Protein Resource (UniProt), based on the functional description available (https://www.uniprot.org, accessed on 13 February 2021). Therefore, the remaining 2275 inherited variants were targeted for further investigation, and 6 of them were selected since they are located in the mitochondria-related genes *POLG, SSBP1, MTCH2, ATAD3A,* and *PAM16,* with a minor allele frequency of <0.03 in the general population (Figure 3B,C). Among these six variants, c.868C>T (p. [Arg290Cys]) within the *POLG* gene was predicted to have some pathogenic effect, such as Leigh syndrome, based on the GATK analysis pipeline (https://gatk.broadinstitute.org, accessed on 13 February 2021) and the highest-rank score for pathogenicity prediction using in silico prediction tools (Figure 3B). 

This variant was reported as a homozygous variant in a 2-year-old PS patient [24]. This patient was diagnosed with infantile spasms and showed multiple clinical symptoms, including myoclonic seizures, tonic-clonic seizures, and a global development delay. However, mtDNA analysis was not performed for this patient. Besides, several reports have demonstrated that various *POLG* mutations are associated with mtDNA-deletion-related diseases, including sensory ataxic neuropathy, dysarthria and ophthalmoparesis (SANDO), and progressive external ophthalmoplegia (PEO) (Table 2). 

The second-highest-rank variant, c.320G>A (p. [Arg107Gln]), in the *SSBP1* gene has been reported as an autosomal dominant mutation associated with optic atrophy disorder and severe and progressive mitochondrial disease manifestations across the full Pearson, Kearns–Sayre, and Leigh syndrome spectrum [17,25,26,27].

Hence, *POLG* c.868C>T (p. [Arg290Cys]) and *SSBP1* c.320G>A (p. [Arg107Gln]) were evaluated as candidates for a pathogenic factor in the PS patient according to the pathogenicity predictions using exome sequencing analysis and previous reports.

### 3.3. Deficient Expression of POLG and SSBP1 Induced by Mutations in POLG and SSBP1 Genes

**Figure 4:**
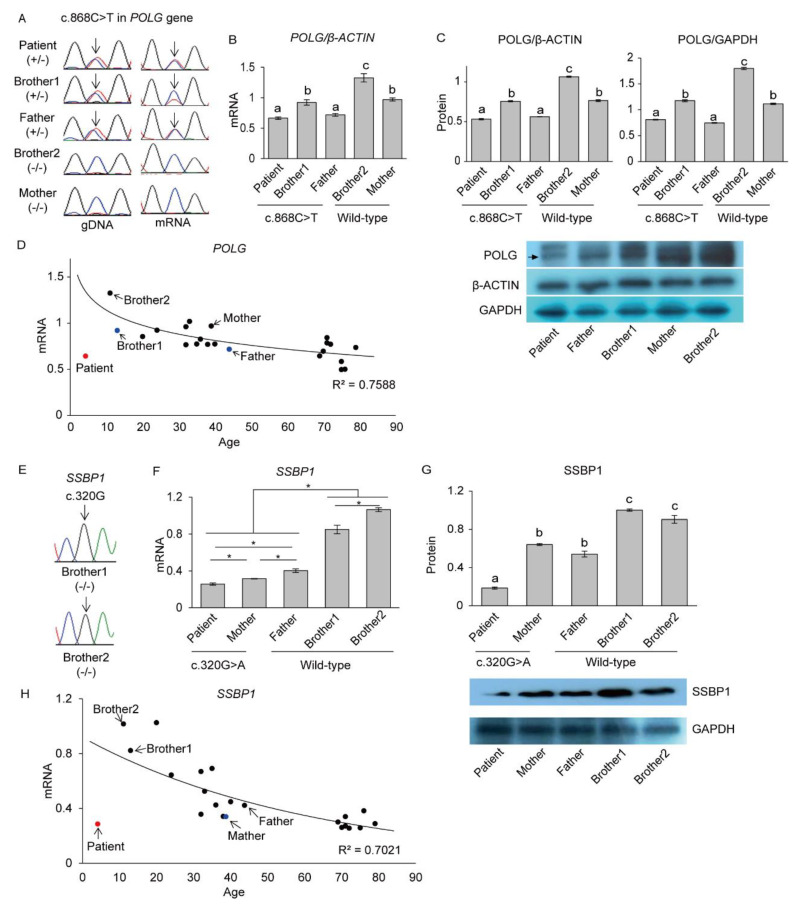
Deficient *POLG* and *SSBP1* expression induced by mutations in the *POLG* and *SSBP1* genes and aging. (**A**) Chromatograph of position c.868 in the *POLG* gene. The PS patient, brother 1, and the father showed a c.868 C>T mutation in both genomic DNA and mRNA. (**B**) Deficient *POLG* mRNA expression induced by *POLG* mutation and aging. The PS patient and carriers showed lower *POLG* mRNA expression than the wild-type group when comparing within similar age of family members (PS patient and brother 1 vs. brother 2; father vs. mother). Further, the father showed lower *POLG* expression than brother 1 in the carrier group, and the mother displayed lower *POLG* expression than brother 2 in the wild-type group. The relative *POLG* mRNA expression was normalized by *β-ACTIN* mRNA. Each assay was completed in triplicate, and the data are represented as the mean ± SEM. The letters a, b, and c indicate significant (*p* < 0.05) differences among family members. (**C**) Deficient POLG protein expression induced by *POLG* mutation and aging. The PS patient and carriers showed lower POLG protein expression than the wild-type group when comparing within similar age of family members (PS patient and brother 1 vs. brother 2; father vs. mother). The father showed lower POLG expression than brother 1 in the carrier group, and the mother displayed lower POLG expression than brother 2 in the wild-type group. The arrow in a blot of POLG indicates the bands that were quantified for relative expression. The relative POLG protein expression was normalized by β-ACTIN and GAPDH protein. Each assay was completed in triplicate, and the data are represented as the mean ± SEM. The letters a, b, and c indicate significant (*p* < 0.05) differences among family members. (**D**) Age-dependent *POLG* expression. Eighteen individuals in the general population and healthy family members of the PS patient showed that *POLG* expression reduces by aging. However, the PS patient displayed lower *POLG* expression compared to others of a similar age. R^2^ means Pearson’s r correlation. Red and blue dots represent the patient and carriers, respectively. (**E**) Chromatograph of position c.302 in the *SSBP1* gene in brother 1 and brother 2. Both had no mutation. (**F**) Deficient *SSBP1* mRNA expression induced by *SSBP1* mutation and aging. The PS patient and his mother, the carriers, demonstrated significantly lower *SSBP1* mRNA expression than the wild-type members, the father, brother 1, and brother 2. The relative *SSBP1* mRNA expression was normalized by *β-ACTIN* mRNA. Each assay was completed in triplicate, and the data are represented as the mean ± SEM; *p < 0.05. Among the healthy family members, the parents showed lower *SSBP1* expression than brothers 1 and 2. (**G**) Deficient SSBP1 protein expression induced by *SSBP1* mutation and aging. The PS patient showed lower SSBP1 protein expression than the healthy family members. The parents had significantly lower SSBP1 protein expression than brothers 1 and 2. The relative SSBP1 protein expression was normalized by GAPDH protein. Each assay was completed in triplicate, and the data are represented as the mean ± SEM. The letters a, b, and c indicate significant (*p* < 0.05) differences among family members. (**H**) Age-dependent *SSBP1* expression. *SSBP1* expression was reduced by aging in the general population. The PS patient displayed lower *SSBP1* expression compared to others of a similar age. R^2^ means Pearson’s r correlation. Red and blue dots represent the patient and carriers, respectively.

A heterozygous c.868C>T *POLG* mutation in the PS patient was confirmed using Sanger sequencing. This mutation was inherited from his father (Figure 4A). Besides, his healthy older brother (brother 1) had the same heterozygous mutation, while the other healthy brother (brother 2) and the mother presented with the wild-type sequence (Figure 4A). Although the same heterozygous mutation (c.868C>T) was shared by the PS patient and two family members (father and brother 1), the mtDNA deletion was only detected in the PS patient. To understand the difference in this disease’s penetrance between the subjects with the *POLG* mutation, the cDNA converted from their mRNA, including the c.868 position, was sequenced using Sanger sequencing. We noted that the wild-type C residue was expressed at lower levels in the PS patient than that observed in brother 1; however, it was expressed at a similar level to that observed in the father (Figure 4A). 

Then the expression of POLG in mRNA and protein levels were evaluated in all family members. The PS patient and the carrier group demonstrated lower *POLG* mRNA expression than the wild-type group (Figure 4B). The highest *POLG* mRNA expression was observed in brother 2, followed by the mother. In an age-grouped comparison, the PS patient and brother 1 showed significantly lower *POLG* expression than that observed in brother 2 and the father displayed lower expression levels than those exhibited by the mother (*p* < 0.05). In addition, in an age-dependent comparison within groups, the father’s *POLG* expression level was significantly lower than that of brother 1 in the carrier group, and the mother’s *POLG* expression level was also lower than that expressed by brother 2. To detect POLG protein expression, two different proteins, β-ACTIN and GAPDH, were used as reference proteins to exclude the possibility of varying reference protein levels caused by aging. β-ACTIN and GAPDH proteins showed a similar expression level in the PS patient and his family members. When POLG protein levels were analyzed using these two reference proteins, POLG protein expression was similar to that of *POLG* mRNA expression (Figure 4C). For confirmation of age-dependent *POLG* gene expression, we evaluated the *POLG* mRNA levels in the general population (18 individuals, 20–79 years old) and healthy family members of the PS patient and found that *POLG* expression was reduced by aging (Figure 4D). The PS patient showed lower *POLG* expression compared to other people of a similar age. Based on these results, it can be assumed that both the mutation in the *POLG* gene and aging could be critical factors in decreased POLG expression.

We also analyzed the expression of *SSBP1* mRNA in the PS patient and his family members. The PS patient and his parents had already been investigated for their genotype in the c.320 position of the *SSBP1* gene in exome sequencing; therefore, we analyzed the same position in brothers 1 and 2 by Sanger sequencing and found that both had no mutation (Figure 4E). The PS patient and his mother, the carriers, demonstrated significantly lower *SSBP1* mRNA expression than the wild-type members, the father, brother 1, and brother 2 (Figure 4F). The SSPB1 protein level was significantly lower in the PS patient than in all other healthy family members (Figure 4G). Among the healthy family members, the parents had significantly lower SSBP1 mRNA and protein expression than brothers 1 and 2 (Figure 4F,G).

Based on these results, *SSBP1* expression was analyzed in the general population, and results showed decreased age-dependent *SSBP1* expression like *POLG* expression (Figure 4H). The PS patient showed lower *SSBP1* expression than other people of a similar age; however, healthy members had comparable *SSBP1* expression (Figure 4H). These results demonstrate that both mutations in the *SSBP1* gene and aging could induce reduced SSBP1 expression.

For the PS patient, the expression of *POLG* and *SSBP1* was significantly lower than other people of a similar age group, which could lead to mtDNA deletion and disease.

### 3.4. De Novo mtDNA Deletions with Methylation Changes 

**Figure 5:**
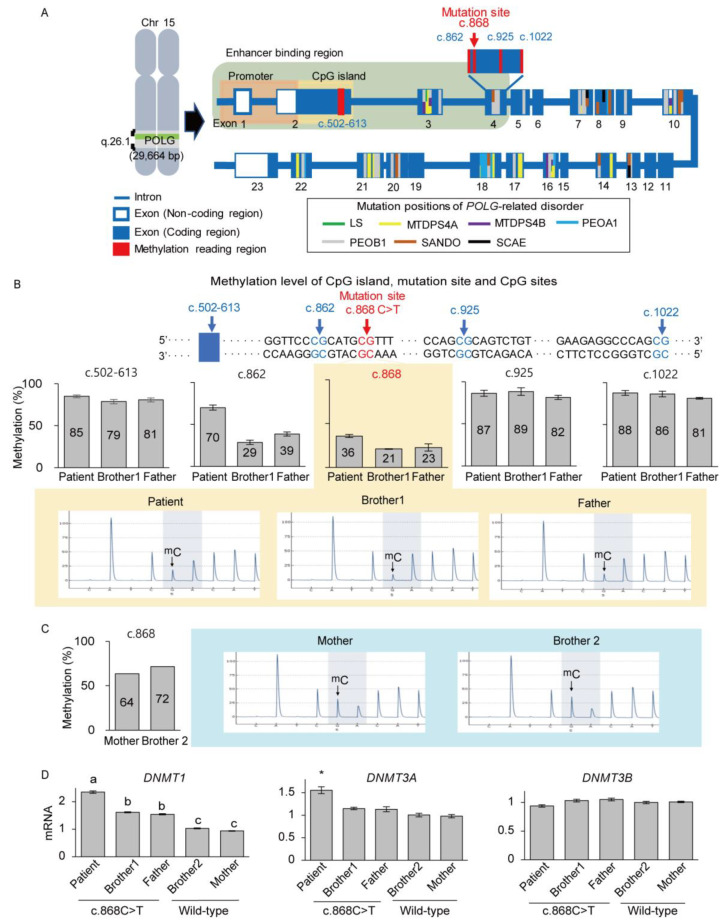
Hypermethylation of c.868 in the *POLG* gene of the PS patient. (**A**) Schematic of the mutation positions of *POLG*-related disorders and methylation readings for various regions of the *POLG* gene. LS, Leigh syndrome; MTDPS4A, mitochondrial DNA depletion syndrome 4A; MTDPS4B, mitochondrial DNA depletion syndrome 4B; PEOA1, progressive external ophthalmoplegia with mitochondrial DNA deletions, autosomal dominant 1; PEOA2, progressive external ophthalmoplegia with mitochondrial DNA deletions, autosomal dominant 2; SANDO, sensory ataxic neuropathy dysarthria and ophthalmoparesis; SCAE, spinocerebellar ataxia with epilepsy. (**B**) The methylation status of CpG islands (c.502–613), mutation position (c.868), and CpG sites (c.862, c.925, and c.1022) are located close to the mutation position in the PS patient, brother 1, and the father. c.862 and c.868 were hypermethylated in the PS patient, while the CpG island and other CpG sites were comparable to those of the wild type. Only the wild-type allele at c.868 could be methylated, with the pyrosequencing results confirming no methylation at the mutated allele at c.868. ^m^C in the pyrosequencing peak map represents a methylated cytosine. Each assay was completed in duplicate, and samples were collected from individual cell culture plates. (**C**) The methylation level of the mutation site in brother 2 and the mother. The assay was performed one time. (**D**) *DNMT* expression levels in the mRNA of the PS patient and his family members. Expression of *DNMT1* and *DNMT3A* was higher in the PS patient compared to that in other family members (*p* < 0.05). Relative *DNMT* mRNA expression was normalized against *β-ACTIN*. The data were normalized with healthy control (brother 2). Each assay was repeated in quadruplicate, and the data are represented as the mean ± SEM; * *p* < 0.05. The letters a, b, and c indicate significant (*p* < 0.05) differences among family members.

We investigated another factor that reduces gene expressions in patients and focused on the degree of methylation in the mutation position in the *POLG* gene for the penetration of pathogenicity. As children, the PS patient and brother 1 harbored the same mutation; however, the PS patient showed a significantly lower POLG expression although PS patient was younger than brother 1. Several CpG sites were selected, including those at the mutation position (c.868) and several other sites (c.502–613, c.862, c.925, and c.1022) in exon 4 of the *POLG* gene (Figure 5A), and analyzed in the PS patient, brother 1, and the father. The methylation status at each site was assessed using the pyrosequencing of the genomic DNA from skin cells. The methylation statuses of c.502–613, c.925, and c.1022 were similar between the subjects (79–89%) (Figure 5B). In the mutation position, c.868C was changed to c.868T in the carriers’ mutant allele. Hence only c.868C could be methylated, and the methylation score predicted by the pyrosequencing reflected that c.868T was not methylated, eventually resulting in a 36% methylation status at position c.868 for the PS patient, while it was 21% and 23% for brother 1 and the father, respectively (Figure 5B). If the methylation score reflects only the methylation status of the wild-type C residue, then the methylation scores could be assumed to be 72%, 42%, and 46%, respectively (Figure 5B). The c.862 position showed that 70% was methylated in the PS patient, while the methylation levels were 29% and 39% in brother 1 and the father, respectively (Figure 5B). The methylation level was measured in wild-type family members, in whom both alleles were C in c.868, resulting in methylation levels of 64% and 72% in brother 2 and the mother, respectively (Figure 5C). Even though the methylation scores of the mother and brother 1 were similar to or less than that of the PS patient, these wild-type family members showed significantly higher POLG expression, suggesting that the methylation level and POLG expression had a different correlation between wild-type and mutated family members. According to the results, the PS patient displayed hypermethylation in the mutation position compared to brother 1, which suggests that the hypermethylation of the wild-type allele could be a critical factor in the penetrance of the mtDNA deletion only in the PS patient.

Next, we evaluated whether the expression of methyltransferases, which regulate methylation of the genome [30], was also different in the PS patient compared to the carriers. The mRNA expression levels of *DNMT1*, *DNMT3A*, and *DNMT3B* were measured by qRT-PCR. The expression levels of *DNMT1*, expected to maintain methylation patterns in newly synthesized DNA during replication, and those of *DNMT3A*, which functions as a de novo methyltransferase [30], were significantly higher in the PS patient than those in brother 1 (Figure 5D). These significant differences were also seen in comparison with other family members. *DNMT3B* levels were similar among all subjects. Taken together, this suggests that hypermethylation in c.868 mutation in the *POLG* gene acts concurrently to support the penetrance of the large mtDNA deletion in the PS patient.

## 4. Discussion

The PS patient harbored multiple mtDNA deletions in several tissues, including the major mtDNA deletion (mt8921–13316), which is a novel mutation that has not been previously reported. This deletion affected mitochondrial function and occurred at various heteroplasmic levels in multiple organs. We hypothesized that the multiple mtDNA deletions in the PS patient could be derived from 5 bp direct repeats in mtDNA deletion based on previous reports that state that mtDNA deletions are often flanked by short direct repeats [10,31]. In the PS patient, a large 10,825 bp deletion (mt3192–14017) was also detected; however, this deletion was not flanked by direct repeats like the major deletion. *POLG* mutations have been related to diseases that involve multiple mtDNA deletions [6,14,28,29]. Even though the father and brother 1 carried the same *POLG* mutation without mtDNA deletion, we concluded that the multiple mtDNA deletions in the PS patient could be related to *POLG* mutation rather than direct repeats. However, the possibility of an association between multiple mtDNA deletions and direct repeats cannot be ignored. 

In the PS patient, we could not find any evidence of maternal inheritance for the major deletion; hence, we evaluated the nuclear factors by performing exome sequencing in the PS patient and his parents. As expected, we were able to identify an inherited variant in the *POLG* gene that could affect mtDNA proofreading. The *POLG* gene variant c.[868C>T] (p. [Arg290Cys]) was identified as having the most potent disease risk following in silico evaluations; this mutation was shown to be paternally inherited, and both his father and his elder brother also presented with this variation. However, the mtDNA deletion occurred exclusively in the PS patient. 

POLG mRNA and protein expression was evaluated to determine the effect of the c.868 mutation. Among the children, the POLG expression in mRNA and protein levels were significantly lower in the PS patient and the carrier (brother 1) than the wild-type child (brother 2). Further, the father, with the *POLG* mutation, also exhibited lower POLG protein expression compared to the wild-type mother. Therefore, we confirmed that this *POLG* mutation reduced POLG expression. Following this, we analyzed *POLG* expression in the general population. The results showed that *POLG* expression decreases with age. Healthy family members of the PS patient showed similar phenomena compared to the general population. On the other hand, the PS patient displayed significantly lower expression compared to others of a similar age. Even though the PS patient and the father showed an equivalent level of POLG expression, only the PS patient had a disease phenotype. We supposed that the threshold of the POLG requirement could be different according to age. Cells of juveniles could require higher POLG expression to avoid pathogenicity, while an adult could demand relatively lower POLG expression. Therefore, only the PS patient had the disease, but the father did not under a similar level of POLG expression.

*SSBP1* also showed age-dependent decreased expression in the general population. We compared *SSBP1* expression in the PS patient and his family to that in the general population. The PS patient showed lower *SSBP1* expression than others of a similar age, like *POLG* expression. Therefore, we concluded that the lower expression of both *POLG* and *SSBP1* genes could contribute to the large de novo mtDNA deletion, leading to the disease in the patient. The PS patient carried mutations in both *POLG* and *SSBP1* genes, while his parents harbored only one of the two mutations without clinical symptoms. A similar digenic case was observed in a PEO patient harboring multiple mtDNA deletions, who carried the co-occurrence of substitution in *POLG* and *C10orf2/Twinkle* [32]. The *POLG* mutation alone in this patient did not cause the clinical phenotype, which was a similar phenomenon to that for one parent of the PS patient.

The PS patient and brother 1 harbored the same mutation; however, only the PS patient showed mtDNA deletion with lower POLG expression than brother 1. There must be additional contributing factors in reducing POLG expression. The methylation of the genome can regulate gene expression [33]; accordingly, we evaluated methylation levels at the mutation sites. As expected, the mutation position was hypermethylated in the PS patient in comparison to brother 1, which could be a critical factor in the induction of lower POLG expression in the PS patient. 

## 5. Conclusions

**Figure 6:**
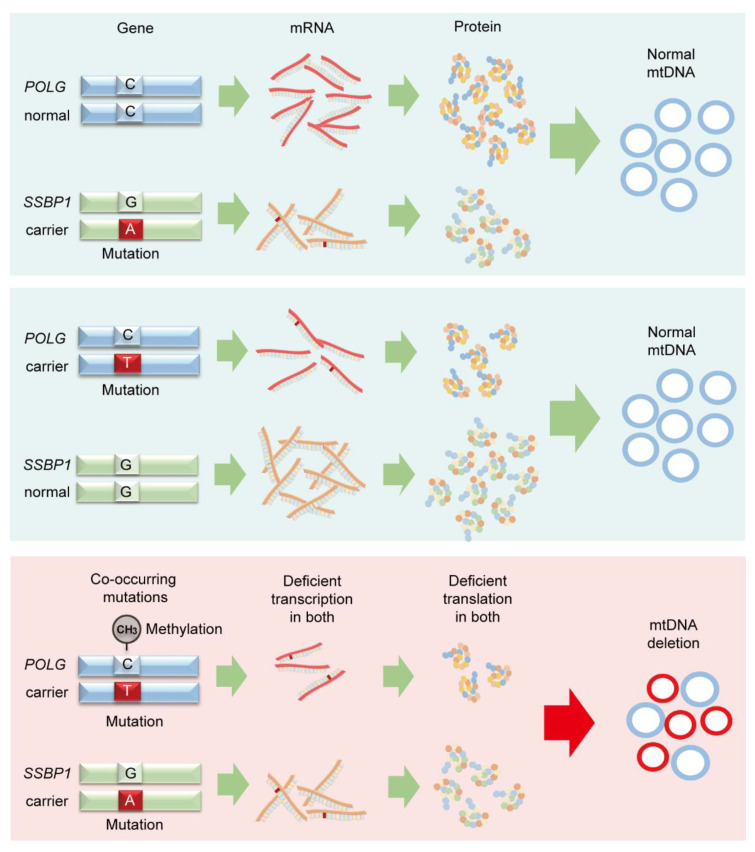
Schema of the penetration of mtDNA deletion and *POLG* and *SSBP1* gene mutations. Mutations in *POLG* and *SSBP1* genes could reduce their expression; however, they did not cause mtDNA deletion independently. Only co-deficient expression of *POLG* and *SSBP1* could induce a large mtDNA deletion. Further, the hypermethylation mutation site could be another critical factor for the penetrance of the mtDNA defect.

Mutations in *POLG* and *SSBP1* genes can negatively affect their mRNA and protein expression (Figure 6); however, they cannot cause mtDNA deletion and related diseases independently. Co-occurring mutations in both *POLG* and *SSBP1* genes lead to deficient expression, which may contribute to the mtDNA deletion development described in this study. Hypermethylation of the wild-type allele within the same region in the *POLG* gene might further destabilize mtDNA integrity. The results suggest that *POLG* and *SSBP1* expression might be associated with the penetrance of mtDNA mutations, but not individually.

## Figures and Tables

**Table 1:** Clinical features at different ages of the PS patient.

Age	Clinical Features
5 months	Sideroblastic anemia Pancytopenia
	Bone marrow failure
2 years	Proximal renal tubular acidosis
3 years	Developmental delay
	Exocrine pancreatic insufficiency
	Adrenal cortical insufficiency
	Chronic kidney disease

**Table 2:** mtDNA deletions associated with *POLG* mutations for various diseases.

Mutation in *POLG* (Allele/Allele)	*POLG* Exon	mtDNA Deletion	Disease/Clinical Features	Reported Year	Reference
p.A467T/p.W748S	7/13	Multiple	Sensory ataxic neuropathy with dysarthria and ophthalmoparesis (SANDO)	2018	[14]
p.P587L + p.T251I/p.R869Q	10+3/17	Multiple		2018	[14]
p.A467Thr/p.A467T	7/7	Multiple		2018	[14]
p.A467Thr/p.R627Q	7/10	Multiple		2018	[14]
p.P648R/p.R807C	10/10	Multiple		2011	[6]
p.Q1236H/wt	23	Single	Inclusion body myositis (IBM)	2015	[28]
p.G451E/wt	7	Multiple	Progressive external ophthalmoplegia (PEO)	2006	[29]
p.M430L/p.W918R	7/18	Multiple		2011	[6]
p.G517V/wt	8	Multiple		2011	[6]
p.W585X/p.P648R	10/10	Multiple		2011	[6]
p.A862T/p.R1081Q	16/20	Multiple	Alpers-like	2011	[6]
p.R1081Q/wt	20	Multiple	Muscle weakness, ataxia, extrapyramidal features, diabetes mellitus	2011	[6]
p.R807C/wt	14	Multiple	Proximal myopathy, ptosis, diplopia	2011	[6]

## Data Availability

The exome sequencing data were deposited in GenBank under accession numbers PRJNA555464.
